# An interdisciplinary course on evolution and sustainability increases acceptance of evolutionary theory and increases understanding of interdisciplinary application of evolutionary theory

**DOI:** 10.1186/s12052-023-00188-4

**Published:** 2023-05-24

**Authors:** Scott A. Kreher, Ellen McManus

**Affiliations:** Dominican University, River Forest, IL 60305 USA

**Keywords:** Evolution acceptance, Interdisciplinarity, Sustainability

## Abstract

**Background:**

Although evolutionary theory is foundational and integrative in modern biology, there remains widespread lack of acceptance among U.S. residents. An interdisciplinary approach to teaching evolutionary theory at the undergraduate level has many advantages, such as giving students a context for learning about evolution and application of evolutionary theory to other academic disciplines and everyday life. While there are foundational examples of interdisciplinary approaches to teaching evolutionary theory, there are few examples of courses with application of evolutionary theory to issues of sustainability, such as conservation or global climate change. We build on the practical and theoretical work of others to create an interdisciplinary course on evolutionary theory for non-science majors, with ties to sustainability. Our course is taught in three modules, with extensive readings and hands-on lab activities. The first module is focused on honey bee biology, with hands-on beekeeping experiences; the second module on native plants and community education on sustainability; and the third module on the evolution of the subjective human experience of free will.

**Results:**

We found that students in our course experienced an increased acceptance of evolutionary theory. We found that students also met the course leaning objectives, of basic knowledge of evolutionary theory and application of evolutionary theory to other disciplines, assessed through group and individual major assignments. We also found that students had an expanded perspective on interdisciplinary application of evolutionary theory, assessed through closed-ended survey questions and analysis of open-ended writing.

**Conclusions:**

Students in our course experienced an increase of acceptance of evolutionary theory and an expanded perspective on interdisciplinary application of evolutionary theory, despite the fact that many students were not science majors.

**Supplementary Information:**

The online version contains supplementary material available at 10.1186/s12052-023-00188-4.

## Background

Evolutionary theory is one of the most important concepts in modern biology and is foundational, integrative, and explanatory. Evolutionary theory is part of curricula for primary and secondary students in many countries, including many regions in the United States (Deniz and Borgerding 2018). The importance of evolutionary theory is underscored by its inclusion as a core concept in the *Vision and Change* report for undergraduate biology education as well as being a core concept in the United States NGSS (Next Generation Science Standards) for K-12 students (American Association for the Advancement of Science 2011; https://www.nextgenscience.org/). Although evolutionary theory is included in many K-12 science curricula and in most undergraduate biology courses, approximately 40% of US adults do not accept evolutionary theory, which suggests a false notion of controversy around a solid body of scientific theory (Funk et al. 2020). A major question for STEM education is how to teach evolutionary theory to all students in a way that increases acceptance of evolutionary theory.

A compelling method to teach evolutionary theory is through an interdisciplinary approach, such as applying evolutionary theory to disciplines outside of biology or an approach centered on complex problems, such as pandemics or climate change, where evolutionary theory could provide explanations and solutions complementary to other ways of thinking (Newell et al. 1990). One reason to take an interdisciplinary approach to evolutionary theory is that it can provide undergraduate non-biology majors with a context for learning about and using evolutionary theory (Benson et al. 2009; Chamany et al. 2008). While evolutionary theory is explored in necessary depth as part of undergraduate biology major curricula, these courses are usually not open to non-biology majors who lack pre-requisite courses. A second reason is that many areas of life outside of the biological sciences, including other academic disciplines, will benefit from better understanding of evolutionary theory. Most aspects of daily life that concern human behavior, such as health care, criminal justice, and economics, will benefit from an evolutionary perspective (Wilson 2007). Additionally, academic disciplines that deal with human nature and behavior will also benefit from interdisciplinary work that incorporates evolutionary theory.

The concept that evolutionary theory can be applied to disciplines outside of the life sciences and to other areas of everyday life has major proponents in the Evolutionary Studies Consortium and the Evolutionary Studies program at Binghamton University-SUNY (Wilson 2005; https://evostudies.org). The Evolutionary Studies Consortium has grown into a network of programs and resources to support interdisciplinary teaching and application of evolutionary theory. The *Evolution for Everyone* course at Binghamton University is a successful example of an interdisciplinary course on evolutionary theory that increases acceptance of evolution (O’Brien et al. 2009). The efforts of educators at Binghamton University and members of the Evolutionary Studies Consortium have led to expanding perspectives on interdisciplinary application of evolutionary theory (Geher et al. 2019).

Hanisch and Eirdosh have built on the foundation of the Evolutionary Studies Consortium and have created learning materials and posed crucial questions related to interdisciplinary application of evolutionary theory (Hanisch and Eirdosh 2020; https://openevo.eva.mpg.de/). Hanisch and Eirdosh have posed theoretical and practical considerations for applying evolutionary theory to sustainability issues, such as conservation, achieving sustainable development, and mitigating global climate change, which is a rational next step in teaching evolution in an interdisciplinary manner (Eirdosh and Hanish 2019). However, there are few examples of courses with application of evolutionary theory to issues of sustainability, accompanied with assessment evidence. As stated in the United Nations Sustainable Development goals, an obvious goal of all biology and evolutionary education should be to promote protection of the environment and conservation of other organisms, both to support human life and for the sake of other organisms in their own right (https://sdgs.un.org/goals). We have built on the work of these and others to create an undergraduate course on evolutionary theory with application to sustainability.

Here we describe our interdisciplinary course on evolutionary theory for undergraduates of all majors, with connections to sustainability, and provide assessment evidence of effectiveness. Our course consists of three modules, each including group or individual projects that illustrate evolutionary concepts as well as sustainability concepts, such as pollinator conservation and community education on sustainability. We assessed our course with the following three research questions:

- Research question 1: Can an interdisciplinary course on evolution, with connections to sustainability, improve acceptance of evolution?
- Research question 2: Do students achieve learning objectives of the course: basic understanding of evolutionary theory and ability to apply evolutionary theory to other disciplines?
- Research question 3: Can an interdisciplinary course on evolution increase understanding of interdisciplinary application of evolutionary theory?

We found that students in our course demonstrated an increase in evolution acceptance and also expanded perspective on interdisciplinary application of evolutionary theory.

## Methods

### Course description

This interdisciplinary course on evolution is co-taught by a biology professor and an English professor at Dominican University (DU), a primarily undergraduate institution that is also a Hispanic-serving institution. The course is taught over a 15-week semester, with online activities and a weekly 3-h in-person session, which meets in a biology lab and sometimes includes visits to a campus native-landscaped area and apiary. The course is housed in the university honors program and is open to any major with junior-level status. The course enrollment is limited to 16, partly because of honors program guidelines but also because of lab seating. The course has been taught in its current form five times, between spring 2015 and fall 2021.

**Fig. 1:**
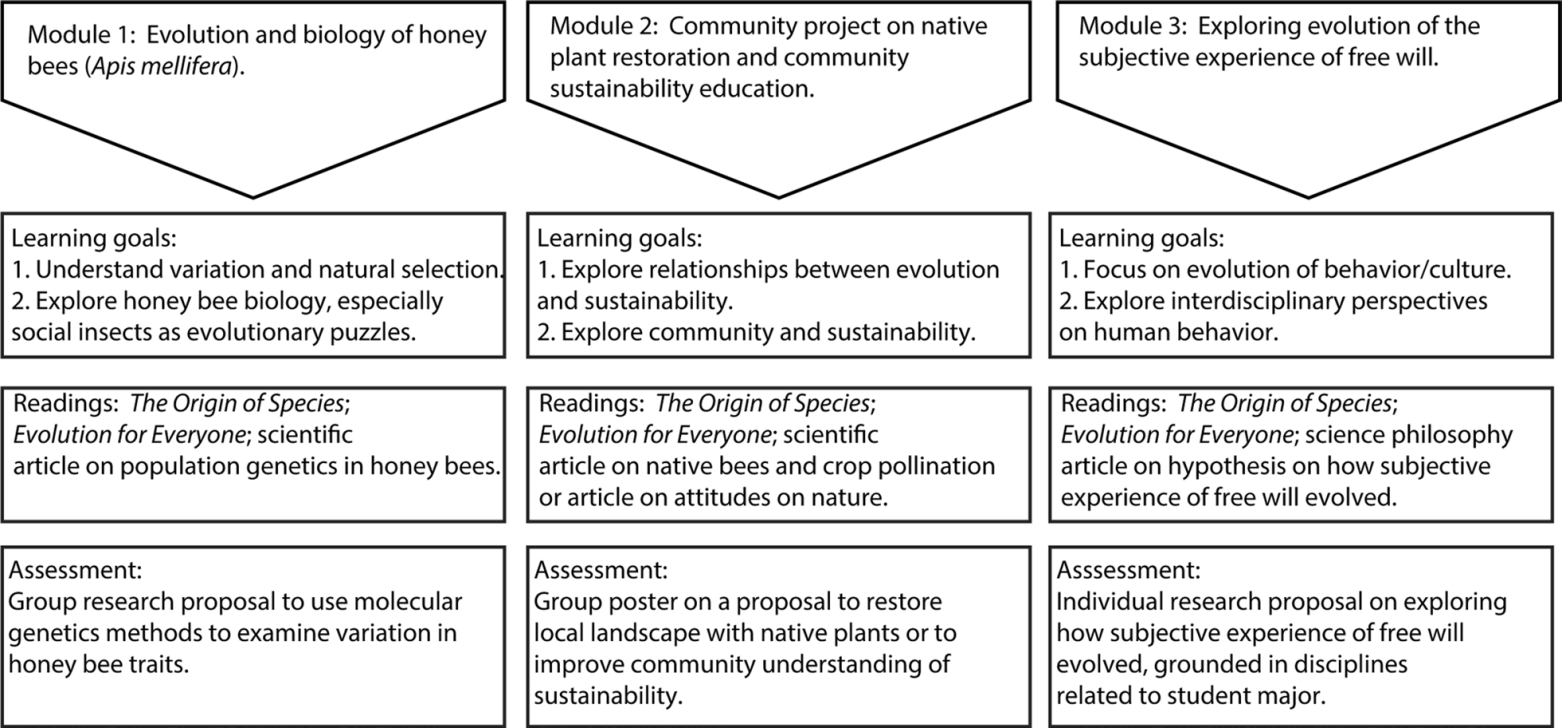
Summary of interdisciplinary course on evolution with connections to sustainability, highlighting the three modules

The course is organized into three approximately five-week modules that build on each other (Fig. 1). The first module focuses on honey bee (*Apis mellifera*) evolution and biology, and its key question concerns social insects as an evolutionary puzzle. The DU campus honey bee hives are central to this module, which includes hands-on bee hive observations and lab work on honey bee variation assessed through DNA techniques, such as PCR on honey bee Variable Number of Tandem Repeat (VNTR) loci and electrophoresis. The concept of sustainability is introduced through readings and activities on pollination and ecosystem services provided by bees. The main assignment of the module is a group research proposal to examine possible relationships between honey bee DNA variation and variation in a relevant honey bee trait, such as foraging, resistance to pesticides, or resistance to parasites, such as *Varroa* mites; the proposal is presented in groups to the class.

The second module’s readings and activities focus on human relationships with the natural world, which students explore primarily through a group project on habitat restoration and/or community sustainability education. Students work in teams to create and present proposals on restoring native plant habitats on the DU campus, in part to support bees and other pollinators, but also to promote awareness about the importance of evolution and coevolution in the health of ecosystems. Of the three modules, the second changed the most over the course of the five semesters, beginning with a focus on creating and then expanding the campus native landscaping and developing into a focus on engaging communities in ecosystem-related sustainability initiatives. While the DU campus has played a central role in most of the second module projects, the emphasis of the assignment has shifted to working with other communities, building on these communities’ culturally-informed relationship with the natural world and specific ecosystems. As students work in groups on their project proposals, the class is visited by university staff and community members with expertise in ecosystem care and community engagement.

The third module returns more explicitly to the idea of evolutionary puzzles, this time in the context of human biology and behavior, focusing on human behavioral plasticity and how the subjective experience of human free will could have evolved. The nature of the evolutionary puzzle is that we may have a subjective experience of free will, because of humans' massive behavioral plasticity, but are also constrained by the need to survive and reproduce; this puzzle is explored in the central article of the module by evolutionary biologist Michael Rose (Rose 2016). The key question here is this: given humans’ enormous behavioral plasticity, what guides us to make choices that promote survival and reproduction? As part of the work of this module, professors from neuroscience/psychology, theology, and philosophy give presentations and engage students in discussions about human behavior and the conundrum of free will. Many of the readings and resources in this module explore how free will is incoherent in many ways and ultimately may not be a helpful way to describe human behavior; please see the Additional file 1: appendix for additional readings. In the central assignment for this module, students are asked to develop a research proposal, ideally from the perspective of their own major discipline, on the interconnections of human behavioral plasticity, the subjective experience of having free will, and the evolutionary processes of survival, reproduction, and adaptation. While the connection to sustainability in this module is more oblique, students are encouraged to contemplate the implications of different human behaviors for the ecological environment, including climate change and ecosystem destruction.

Course readings are substantial and closely integrated with discussions, activities, and major projects. The central readings, which provide the throughline connecting the three modules, are selected chapters from Darwin’s *The Origin of Species* and *The Voyage of the Beagle*, selected chapters from David Resnick’s *The Origin Then and Now*, which provides updated commentary on Darwin, and the entirety of David Sloan Wilson’s *Evolution for Everyone,* a distinctively student-friendly book that is a product of the Evolutionary Studies program at Binghamton University-SUNY (Darwin 1859; Darwin 1889; Reznick 2011; Wilson 2007). In addition, for each module, students read an academic article related to the topic of the module. For the first module, students read an article on honey bee population genetics (Estoup et al. 1994) and selected chapters from *Biology of the Honey Bee* (Winston 1987); the second module features either an article on native pollinator effectiveness or articles about community epistemologies of nature and science and the roles that *Homo sapiens* has played in maintaining healthy ecosystems (Bang and Medin 2010; Garibaldi et al. 2013; Root‐Bernstein and Ladle 2019); for the third module, students read an article by evolutionary biologist Michael Rose, published in the 2016 collection *Darwin’s Bridge: Uniting the Humanities and Sciences.* In this article, Rose presents a speculative hypothesis about how, despite extensive behavioral plasticity and subjective experience of free will, the human species still seems to respond to the evolutionary imperatives of survival, reproduction, and care for offspring (Rose 2016). Finally, for each module students read a short story or excerpts from a novel that explores the themes of the module, for example Ian McEwan’s *Enduring Love* for the first module, E.O. Wilson’s *Anthill* for the second, and John Green’s *Turtles All the Way Down* for the third module.

Course activities take place both online and in class. Throughout the week students engage in online discussions of the readings and the upcoming major assignments. They take an individual or group quiz on the readings each week, either online or at the beginning of the weekly in-person class meeting. During the class meetings students may also engage in further discussion of readings, perform lab or field work, hear from guest speakers, work in groups on the major projects, and/or give presentations on these projects.

Please see Additional file 1: appendix for more course details.

### Participants

Students in the course are members of the university honors program and have a variety of majors across all disciplines. The course is not housed in a science department, although approximately 56% of students taking the course have been science majors. The course fulfills an “Explorations and Investigations” requirement of the honors program. Most students are in their third undergraduate year. There have been 66 total students enrolled in the five instances of the course. 80% of the students were female. 39% of students were Latinx/Hispanic backgrounds; 58% of students were white; 3% of students were Asian; the composition of the course reflects overall student composition of the institution.

### Data collection

Data were collected from all students in the first week of classes (pre) and the final week of classes (post). This study was approved by the DU IRB (IRB #2017-260). Student data were collected and pooled from five substantially similar instances of the course, beginning in spring 2015 and ending in fall 2021. All data were included from consenting students with complete pre and post data.

We took a mixed methods approach, with closed-ended instruments and analysis of qualitative data. Collected data consisted of the Generalized Acceptance of Evolution Evaluation 2.1 (GAENE), a validated instrument on evolution acceptance consisting of 13 questions with five answer categories; scores can range from 13 (low evolution acceptance) to 65 (highest evolution acceptance) (Glaze et al. 2020; Smith et al. 2016).

We also collected data on interdisciplinary perspective on evolution, through 3 closed-ended questions that we formulated; scores could range from 3 (reduced perspective) to 15 (increased perspective); see Additional file 1: appendix for questions. Open-ended written answers were collected on three themes: first, written explanations of evolutionary theory, collected pre and post; second, explanations of interdisciplinary application of evolution, only collected post; third, written descriptions of how evolutionary theory could affect future careers and lives, only collected post. See Additional file 1: appendix for open-ended questions.

### Data analysis

Closed-ended GAENE 2.1 scores and closed-ended interdisciplinary perspective on evolution scores were tested for pre to post differences using the nonparametric paired Wilcoxon test.

For analysis of open-ended writing, answers were coded in a first round and then codes were grouped in a second round. Codes were scored as present or absent. All open-ended answers were independently coded by the two authors and subsequently discussed. For research question 2, the open-ended question on explanation of evolutionary theory, first-round codes of natural selection and adaptations were grouped into the second-round code ‘mechanism;’ first round codes of environmental change, changes in populations, similarity of existing species, and divergence of species were grouped in the second-round code ‘phenomenon.’ For the ‘teleological’ code, any written answer that demonstrated purpose in evolution beyond survival or reproduction was coded positive.

For research question 3, answers were coded as phrases for round 1 and grouped into themes for round 2.  Answers were only collected post.

Frequency of codes was determined for open-ended questions. Paired pre-post code frequencies were compared using McNemar’s test.

All statistics were conducted with R.

## Results

### Research question 1: can an interdisciplinary course on evolution, with connections to sustainability, improve acceptance of evolution?

**Fig. 2:**
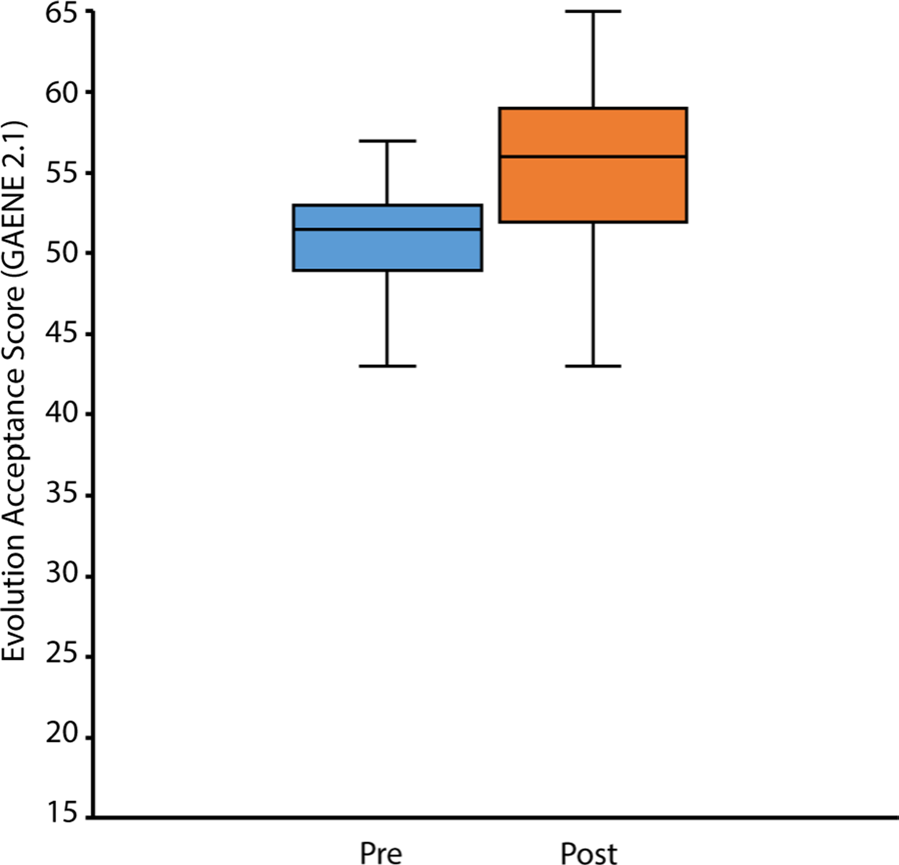
Evolution acceptance increases during the course. Evolution acceptance measured using GAENE 2.1., pre versus post. Scores can range from 13 (lowest acceptance) to 65 (highest acceptance). Pre versus post scores were significantly different (Paired Wilcoxon test, p < 0.01). n = 48 students

We found that while acceptance of evolutionary theory was relatively high in students entering our course, acceptance did increase by course end. We examined acceptance of evolution in connection with the course by using the Generalized Acceptance of Evolution Evaluation 2.1 instrument (GAENE) (Smith et al. 2016). GAENE 2.1 scores were collected in the first week of classes (pre) and in the final week of classes (post) (Fig. 2). The median pre score, 51 of a possible range of 13–65 (65 indicating highest evolution acceptance), was moderately high and suggested a relatively high level of evolution acceptance, raising the possibility of ceiling effects (n = 48 students). However, there was a statistically significant increase of the evolution acceptance score, with a median post score of 56 (paired Wilcoxon test, p < 0.01), indicating that while the pre score was high, evolution acceptance did increase over the course. The median gain (paired post score—pre score) was 4, with 40/48 students having a gain above 0, indicating that most students experienced an increase in acceptance of evolutionary theory.

While our course was open to all majors (there are no science course pre-requisites) we did have many science majors as students (56% of all enrolled students). In order to further explore how experiences in the course were associated with changes in evolution acceptance and associated with previous course experience, we examined how number of science courses taken at the undergraduate level was associated with pre and post evolutionary acceptance scores. We found a statistically significant relationship between self-reported number of undergraduate science courses and the pre- evolutionary acceptance scores, where students with more undergraduate science courses tended to have higher levels of acceptance (Kruskal–Wallis test, Chi-square value = 13.594, df = 5, p-value = 0.0184). Interestingly, the post- evolutionary acceptance scores were not statistically associated with self-reported number of undergraduate science courses (Kruskal–Wallis test, Chi-square value = 5.3303, df = 5, p-value = 0.3769). This evidence does suggest that experiences in the course may be associated with increases in evolution acceptance. We found no relationship between number of secondary science courses and pre- or post- evolution acceptance values, but there was much less variation in number of secondary level courses taken.

### Research question 2: do students achieve learning objectives of the course: basic understanding of evolutionary theory and ability to apply evolutionary theory to other disciplines?

**Fig. 3:**
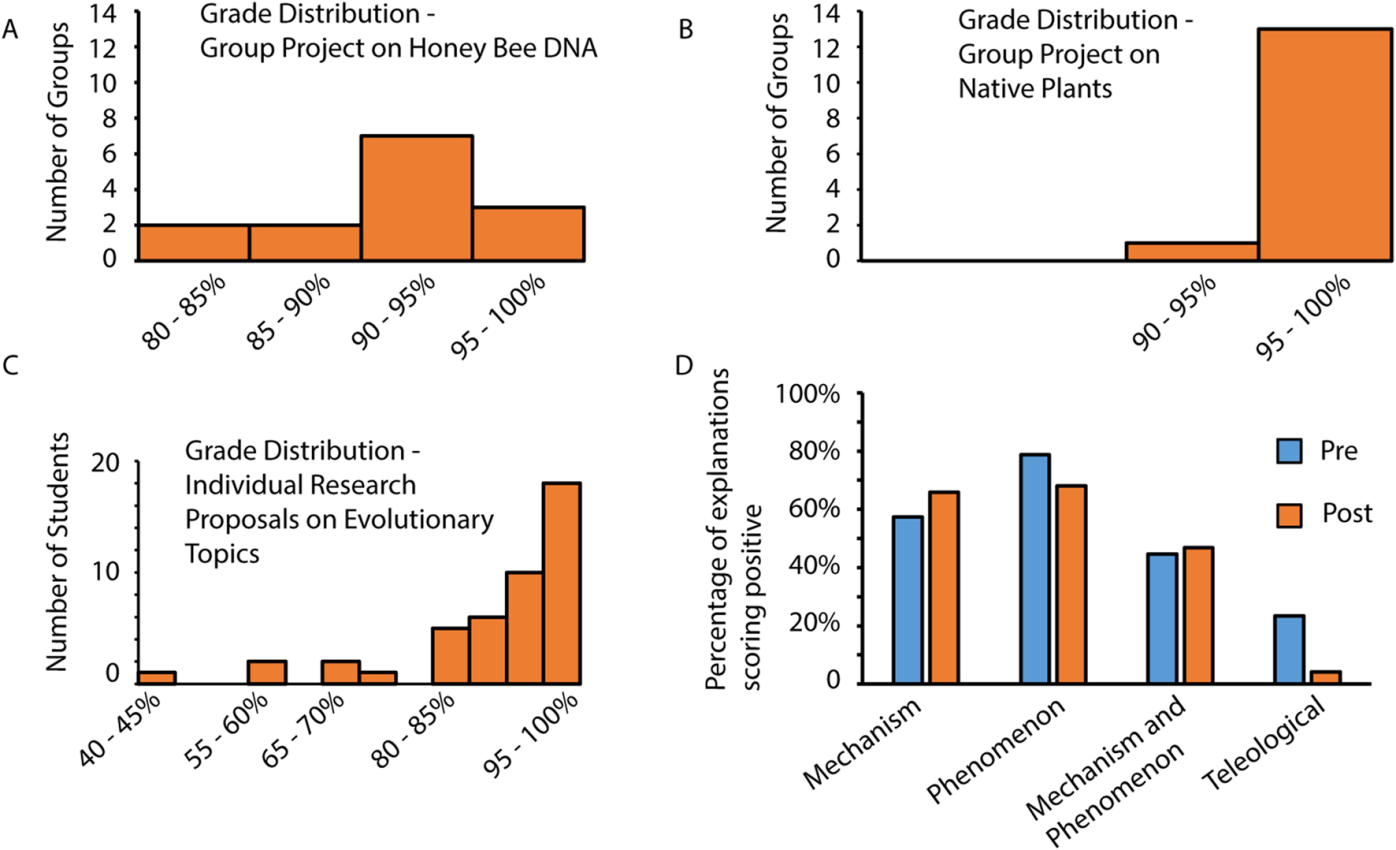
Students successfully achieved course learning objectives. **A** Grade distribution for group research proposals to examine how variation in honey bee DNA can be analyzed in relation to relevant phenotypes, such as colony survival or resistance to pesticides. Average group score = 92%. n = 14 groups. **B** Grade distribution for group poster presentations on proposals to improve native plant habitats of locations on the DU campus or proposals to include community in sustainability education initiatives. Average group score = 99%. n = 14 groups. **C** Grade distributions for individual research proposals to examine an evolutionary topic in depth. Average individual score = 89%. n = 45 students. **D** Quality of individually written explanations of evolutionary theory. Written explanations of evolutionary theory were collected pre and post and coded to determine presence or absence of themes. There were no significant statistical changes in frequency of themes in explanations by McNemar’s test for mechanistic or phenomenological explanations, although percentage of answers containing teleological reasoning decreased significantly from pre to post (McNemar’s test; Chi-square value 9.1; p = 0.003). n = 48 students

We measured achievement of course learning objectives, basic understanding of evolutionary theory, and ability to apply evolutionary theory to other disciplines, through three major assignments: a group presentation of a research proposal on examination of variation in honey bee DNA, particularly related to pollinator conservation; a group poster presentation on a community-based proposal to improve native plant habitat or engage the community in a sustainability-related project; and an individual written research proposal on how the subjective experience of human free evolved and could be adaptive (Fig. 3). Each major assignment assessed course learning objectives; for a detailed description of all course learning objectives please see appendix.

We found that students achieved learning objectives, as measured through grades on the group project on honey bee DNA variation (average group score = 92%; Fig. 3A) and on the group project on native plants and community-based sustainability education (average group score = 99%; Fig. 3B). We note the very high scores for the group project on native plants and through student comments we know that this project was extremely engaging for students. There was much more variation in the individual written research proposals (Fig. 3C); however the average score of 89% was high. Thus we conclude through our major assignments that students largely met course learning objectives.

We additionally measured basic understanding of evolutionary theory through coding of written explanations of evolutionary theory that were collected from students pre and post (Fig. 3D). On the pre survey, 57% of explanations contained mechanistic statements, such as explanations of natural selection or adaptations; 79% of explanations contained a reference to phenomena explained by evolutionary theory, such as relatedness of species or changes in populations over time; 45% of explanations contained both mechanistic and phenomenological statements (n = 48 students) (Fig. 3D). The frequencies of positive-scoring written explanations of evolutionary theory were moderately high in the pre-answers, indicating that students had fairly good understanding of basic elements of evolutionary theory upon entering the course, raising the possibility of ceiling effects; we note that our course was part of the university honors program. However, despite the observation that acceptance of evolution scores increased from pre to post, there were no significant changes in code scores of written explanations of evolutionary theory (McNemar’s test; p > 0.05), with one exception: fewer post explanations contained teleological reasoning. On the pre survey, 23% of explanations contained teleological statements of some type, such as claiming that species evolve in order to become more complex or more intelligent; only two of 47 post explanations (4%) contained teleological statements; this change was statistically significant when corrected for multiple comparisons (McNemar’s test; Chi-square value 9.1; p = 0.003) (Fig. 3D).

### Research question 3: can an interdisciplinary course on evolution increase understanding of interdisciplinary application of evolutionary theory?

**Fig. 4:**
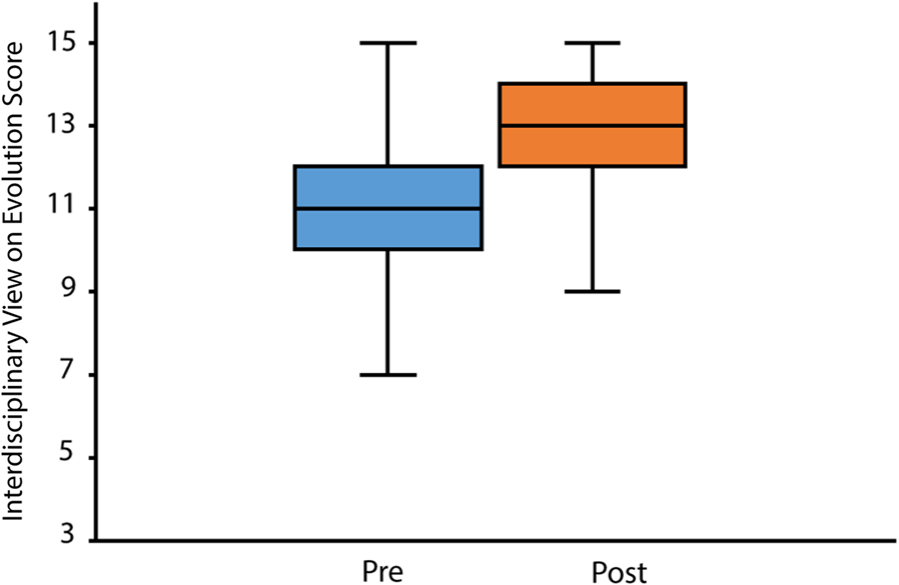
Perspective on interdisciplinary application of evolutionary theory expands over course. Perspective score was measured through a three-question set on applicability of evolutionary theory to other disciplines or everyday life. Scores ranged from 3 (low perspective) to 15 (highest perspective). Pre versus post scores were significantly different (Paired Wilcoxon test, p < 0.01). n = 48 students

We did find that understanding of interdisciplinary application of evolutionary theory increased in students taking our course. We analyzed perspective through two pieces of evidence: first, we used a set of closed-ended questions to create an interdisciplinary perspective score; second, we analyzed written responses to two open-ended questions. We crafted a set of three closed-ended questions to analyze perspective on interdisciplinary application of evolutionary theory, where scores could range from 3 (lowest perspective) to 15 (highest perspective) (Fig. 4). Pre scores were relatively high, with a median value of 11. Post scores had a modest but statistically significant increase, to a median value of 13 (n = 48 students; Paired Wilcoxon test, p < 0.01), indicating that students had an expanded  perspective on application of evolutionary theory to other disciplines or to everyday life.

As an additional form of evidence about student perspective on interdisciplinary application of evolutionary theory, we also analyzed written responses to two open-ended questions, included only in the post survey (Table 1). First, we asked students: *Can you think of any ways in which an understanding of evolution might help us address twenty-first century social or environmental problems*? We coded written responses and analyzed frequency of themes. 34.8% of answers contained the broad theme of how evolutionary theory might allow us to more insightfully reflect on human nature. 19.6% of answers included statements that evolutionary theory may promote more support for conservation and preservation of nature, which was a frequently-referenced theme of the course. Finally, 17.4% of answers stated that evolutionary theory may allow a better understanding of how humans operate in groups, which was a prevalent theme in one of the central course readings, *Evolution for Everyone* (Wilson 2007).

**Table 1:** Student perspectives on interdisciplinary application of evolutionary theory

Can you think of any ways in which an understanding of evolution might help us address twenty-first century social or environmental problems?			
Themes	Allows self-reflection on human nature	May increase support of conservation	Allows better understanding of group behavior
Percentage of written answers with themes	34.8%	19.6%	17.4%

Can you think of any ways that your understanding of the significance of evolutionary theory might help shape the kind of work you do in the future or the way that you live your life?				
Themes	Increases knowledge of human behavior	Application to medicine	Application to environment / sustainability	Application to nutrition
Percentage of written answers with themes	28.3%	23.9%	8.7%	6.5%

Written answers were coded from two open-ended questions on how evolutionary theory might help address social and environmental issues or how evolutionary theory could affect future careers or lives. Written answers were coded and summarized; most frequent codes are displayed. n = 48 students

The second open-ended question included only in the post survey was: *Can you think of any ways that your understanding of the significance of evolutionary theory might help shape the kind of work you do in the future or the way that you live your life*? (Table 1). 28.3% of answers referred to possible application to human behavior, possibly reflecting interests in psychology, medicine, and education. 23.9% of answers contained statements that evolutionary theory could help careers in health and medicine, reflecting the interests of life sciences majors in the course. Some students included statements about how evolutionary theory could be applied to environmental protection or sustainability (8.27% of answers). Because this open-ended question elicited answers about more personal aspects of life, the answers regarding environmental protection and sustainability could reflect new or internalized values that resulted from the course; since we collected these statements only post course, it’s hard to know what type of change, if any, these answers may represent. Answers also included possible application to nutrition (6.5% of answers), and some rare answers mentioned application of evolutionary theory to art and family planning.

## Discussion

We found that students who complete our interdisciplinary course on evolution, with ties to sustainability, experienced an increase of acceptance of evolutionary theory. Evolutionary theory, along with other broad areas of knowledge and belief, such as awareness of global climate change and understanding of causes and effects of viral pandemics, is strongly supported by scientific evidence; yet there is wide variation in acceptance globally outside scientific communities. In the United States, approximately 60% of adults accept evolutionary theory, which has been a stable percentage over twenty years, meaning that a significant 40% of US adults do not accept evolutionary theory (Cooperman, Alan et al. 2013; Funk et al. 2020). It’s difficult to conclude that experiences in our course were causal for increased acceptance of evolution because we do not have quasi-experimental data comparing control courses on evolutionary theory to our course. An alternative explanation is that experiences in any course on evolutionary theory would lead to increased acceptance. However, other researchers have reported that evolutionary acceptance does not increase on average for all evolutionary biology courses (Lindsay et al. 2019). A useful approach would be to measure changes in evolution acceptance in different student populations with a similar course.

One simple hypothesis is that acceptance of evolution is tied to understanding of evolutionary theory—people who do not understand evolutionary theory might have low acceptance, and thus better biology education may increase acceptance. However, the evidence supporting the hypothesis that acceptance of evolutionary theory is tied to knowledge is mixed, and there is evidence that some people with high scientific knowledge have views that diverge from scientific consensus (Drummond and Fischhoff 2017; Dunk et al. 2019). Evidence is emerging that other factors may be more explanatory for acceptance of evolutionary theory: people with high religiosity tend to have lower evolutionary acceptance; people with certain political views also tend to have lower evolutionary acceptance (Drummond and Fischhoff 2017; Funk et al. 2020; Paz-y-Miño, Guilermo and Espinosa 2015).

An emerging hypothesis in biology education is that culturally-relevant teaching can improve acceptance of evolution, especially relative to student identity (Barnes and Brownell 2017). In one study, a course that acknowledged students’ religious identity found increased acceptance of evolution (Lindsay et al. 2019). In a second study, framing evolutionary theory as related to public health was found to increase acceptance (Stover et al. 2013). While we did not deliberately seek to enact culturally-relevant pedagogy, we may be increasing acceptance of evolution through similar mechanisms often grouped under the concept of constructivist pedagogy (Fosnot 1996): group learning (through online discussions, group quizzes, and group projects); hands-on learning with socially and personally meaningful goals; embedding of learning in complex contexts (guest speakers with varied expertise, stakeholders as audiences for presentations, connection of assignments to campus and community history and needs); and discussion of evolution in historical, personal, social, cultural, and wide-ranging scientific contexts. We note the very high grades on the group poster presentations on proposals to develop native plant habitats on our campus or to educate the community on sustainability (Fig. 3B). Student feedback and engagement were overwhelmingly positive for this module and could represent strong positive outcomes predicted from constructivism.

Our course was notably not a science course per se and could be seen as a model for general undergraduate education. Introductory undergraduate biology courses, even if two semesters, often include only a few weeks of direct instruction on evolution, though this can vary considerably. If the understanding and acceptance of evolution were a goal of general undergraduate education, modules similar to those in our course could easily be integrated into both science and non-science undergraduate courses with a wide range of overall themes and focuses, adapted by other institutions.

At the same time, the approach used in our course might also enhance understanding and acceptance of interdisciplinary teaching and learning. Whereas increased acceptance of evolution has been our primary goal, a related goal is to enhance students’ understanding of the multidisciplinary dimensions of evolutionary theory and application and perhaps, as a corollary, their appreciation of interdisciplinary approaches to understanding the world and solving complex problems. We did gather some evidence that students who completed our course had a broader view of possible applications of evolutionary theory. For example, scores on the interdisciplinary application of evolutionary theory survey increased pre to post; we also saw common, positive themes emerge from student answers to open-ended questions about applications of evolutionary theory, especially related to sustainability and conservation. However, we need improved tools to assess how students experience scientific concepts in interdisciplinary ways. For example, an interesting hypothesis for further study is that people who have greater acceptance of evolution might also place a greater value on conservation of nature and on the existence of natural systems for their own sake, independently of value to humans. Another interesting focus for future study is how people view their relationships with nature. Are people with greater acceptance of evolution more likely to view humans as part of nature and interdependent with it? These questions should be addressed in the evolution education community, especially among people who are interested in interdisciplinary approaches to teaching about evolution and people interested in robust, culturally-relevant, and interdisciplinary approaches to teaching about climate change and sustainability.

Having longitudinal data from students who have completed courses such as ours would be useful for answering these questions. However, some evidence may already have emerged from the COVID-19 pandemic. In an analysis of student attitudes and behaviors regarding COVID-19, researchers found that higher levels of knowledge and specific attitudes, such as positive views of science and skepticism of misinformation, were strongly associated with positive public health behaviors and stated willingness to support future societal pandemic prevention measures (Herman et al. 2022). This is promising evidence that contextually meaningful scientific knowledge and attitudes are associated with pro-social behaviors, which perhaps could include support of global sustainability goals. Our course builds on the work of others teaching evolution in an interdisciplinary way, and is in the spirit of Gormally and Heil, who recommend that educators take deliberate steps to better educate and include non-science majors in courses to improve scientific literacy (Geher et al. 2019; Gormally and Heil 2022; Hanisch and Eirdosh 2020). A major recommendation of Gormally and Heil is that science be taught to non-majors in a contextualized way with a community focus, which is a goal of our course.

Finally, a question has been forming in our minds over the last few years and thus is not reflected in our data but might be of interest to future interdisciplinary researchers or teachers of interdisciplinary courses on evolution: Is the human destruction of the biosphere itself an evolutionary puzzle? The concept of the evolutionary puzzle, which we borrow both from David Sloan Wilson and from Darwin himself, refers to a trait that does not seem to benefit the individual but which has nonetheless spread within and appears to be adaptive for a population. The altruistic sacrifice of individual reproduction by eusocial insects is the example that Darwin famously explores in Chapter 7 of *The Origin* (Darwin 1859). An example that Wilson explores is pregnancy sickness, which appears to be maladaptive for reproduction but on closer examination turns out to help protect the developing embryo (Wilson 2007). Although Michael Rose, in his 2016 article featured in our third course module, does not explicitly refer to this concept, in his focus on humans’ behavioral plasticity, large and expensive brains, and subjective experience of free will, he is clearly aiming to untangle some daunting conundrums of human evolution (Rose 2016).

The concept of the evolutionary puzzle is a productive one for teaching and learning, particularly for interdisciplinary teaching and learning, because it encourages thinking outside the disciplinary box, and our course uses it effectively in the first and third modules, in which it helps students explore, respectively, the tension between individual and group survival and the risks and benefits of behavioral plasticity. Although the second module, focusing on human interactions with ecosystems, links the other two, we did not, at first, frame it in terms of an evolutionary puzzle. But as we continued teaching the course and developing the second module more in terms of human interactions with and attitudes toward ecosystems—and, frankly, as the environmental crisis intensified and students’ awareness of it grew—we began to make deeper connections between the evolutionary puzzles of the first and third modules—individual and group survival; the complex implications of human behavioral plasticity—and the phenomenon explored in the second module, how humans interact with ecosystems. Is human behavioral plasticity and growing ability to manipulate other living systems a trait that seems maladaptive but in fact promotes group survival and adaptation, or is it a trait that seems adaptive but in fact threatens long-term group survival, making it a whole new kind of evolutionary puzzle? The question is further complicated by the fact that human cultural practices vary widely in terms of emphasizing cooperation with or manipulation of other living systems.

## Conclusions

Students in our course experienced an increase of acceptance of evolutionary theory and an expanded perspective on interdisciplinary application of evolutionary theory, despite the fact that many students were not science majors. Our course, an interdisciplinary approach to evolutionary theory with connections to sustainability, is an additional example of how to teach evolutionary theory to all students through engaging and flexible modules that could possibly be adapted by other institutions. The course modules, readings, and topics also have connections to students’ communities and everyday lives, and increase of acceptance of evolutionary theory and expanded interdisciplinary perspective may be explained through the constructivist theory of learning.

## Supplementary Information

**Additional file 1. Appendix.** I. Course learning objectives. II. Open-ended question to examine written explanations of evolutionary theory. III. Closed-ended questions of interdisciplinary perspective on evolution. IV. Open-ended questions to examine interdisciplinary perspective on evolutionary theory. V. Major Assignments.

## Data Availability

All data and instruments will be available upon request.
