# Supplementary material for: An interdisciplinary course on evolution and sustainability increases acceptance of evolutionary theory and increases understanding of interdisciplinary application of evolutionary theory
Source: Evolution (N Y). 2023 May 24;16(1):7. doi: 10.1186/s12052-023-00188-4 (PMC10206573; doi:10.1186/s12052-023-00188-4)
Supplement: Supplementary file 1 — Additional file 1. Appendix. I. Course learning objectives. II. Open-ended question to examine written explanations of evolutionary theory. III. Closed-ended questions of interdisciplinary perspective on evolution. IV. Open-ended questions to examine interdisciplinary perspective on evolutionary theory. V. Major Assignments. [file 12052_2023_188_MOESM1_ESM.docx]

Kreher and McManus Appendix

I. Course learning objectives:

- Through reading and discussing chapters of *On the Origin of Species* and excerpts from *The Voyage of the Beagle*, acquire a basic understanding of how Darwin developed his theory of natural selection and presented it in writing.
  - Specific learning objectives include demonstrating understanding of natural selection as a mechanism of evolution that can lead to adaptations. Demonstrating understanding of how evolutionary theory can explain and unite key phenomena, such as similarity of existing species, how species change over time, and how populations change over time.
- Through reading and discussing *Evolution for Everyone*, acquire a well-informed awareness of and curiosity about the history and current status of evolutionary thinking, research, and debate.
- Through weekly activities in class, acquire a practical, hands-on understanding of how evolution happens and begin developing skills in asking questions about evolution and designing experiments, observations, and other kinds of inquiry to answer those questions.
- Through readings, discussions, assignments, and work with Dominican University's honeybee project, explore what honeybees can teach us about evolution, biodiversity, and human behavior.
- Through a deeper exploration of one aspect of human nature--human behavioral plasticity and subjective experience of free will--be able to develop and present in writing a proposal for an experiment, observation, or other form of inquiry, perhaps related to your field of study, to probe a hypothesis about the evolution of that aspect of human nature.
- Develop your skill in the kinds of reading, thinking, observing, experimenting, analyzing, discussing, writing, critiquing, collaborating, and teamwork required in college and beyond.
- Develop your own well-informed (if perhaps tentative) position on the role of evolutionary thinking across the disciplines.

II. Open-ended question to examine written explanations of evolutionary theory:

In your own words, and to the best of your ability, explain the meaning of the theory of evolution.

III. Closed-ended questions on interdisciplinary perspective on evolution

1. Understanding evolution helps me make sense of my everyday life.

Strongly agree

Agree

Disagree

Strongly disagree

I don’t know

1. Evolutionary theory can be applied to many disciplines beyond biology.

Strongly agree

Agree

Disagree

Strongly disagree

I don’t know

1. Although there are many academic disciplines and ways of thinking, knowledge about the world can be ultimately merged into a continuous and unified whole.

Strongly agree

Agree

Disagree

Strongly disagree

I don’t know

IV. Open-ended questions to examine interdisciplinary perspective on evolutionary theory:

1. Can you think of any ways in which an understanding of evolution might help us address 21st century social or environmental problems?
2. Can you think of any ways that your understanding of the significance of evolutionary theory might help shape the kind of work you do in the future or the way that you live your life?

V. Major Assignments:

# Assignment 1: Group Presentation of a Proposal for a Research Project Using DNA Microsatellite Analysis to Answer a Question Related to Honey Bee Behavior or Honey Bee Health

Assignment 1 is a group presentation of a proposal for a research project using DNA microsatellite analysis to answer a question related to honey bee behavior or honey bee health.

There are two things that we very much want you to learn from this assignment:

1.  To think carefully about how evolutionary theory, knowledge about the processes of natural selection, and a basic understanding of how to conduct DNA research using microsatellite analysis might help you answer interesting and/or urgent questions about honey bee behavior or honey bee health.

2. To explore through experience how to present research or a research proposal by identifying a question, framing it in terms of existing knowledge and ideas, describing research materials and methods, and discussing alternatives and implications.

(We will explore the missing steps, presenting and discussing results, through reading and discussing academic articles.)

The Assignment

Working with your team, choose one of the topics from the list below, or a variation on one of them; identify a question about that topic that might be answered through DNA analysis, based on evolutionary theory and the processes of natural selection; frame this question within the theories, ideas, research, evidence, and unanswered questions that we have encountered so far in the course (see the list of required and possible sources below); drawing on the Estoup article and your own experiences with microsatellite analysis, and in consultation with the course instructors as necessary, describe the materials, methods, and procedures that you would use to answer this question; and then discuss then discuss what you expect to find, possible alternative or unexpected findings, and the implications of these expectations and possibilities.

Create a PowerPoint presentation of about 15-20 slides in which you "pitch" this proposal as convincingly as possible to your audience.  Rehearse the presentation thoroughly until you can, as a team, present it effectively in 15-20 minutes.  Deliver the presentation in class.

Materials and Guidelines

Here are the possible topics.  All are related to honey bees, of course.

- Division of Labor
- Foraging Behavior
- Swarming, Scouting, and New Hive Choice
- Learning and Memory
- Anti-Social Behavior
- Dance Language
- Varroa Mite Management
- Susceptibility to Pesticides (e.g., Neonicotinoids)
- Preference for and Susceptibility to Caffeine
- Winter Dead Outs
- Minnesota Hygienic Queens

Here are the sources that you must include in framing your question (that is, in your literature review).  Please note that although we have listed all the chapters in Darwin, Wilson, and Winston covered so far, you may choose your source materials from among any of these chapters.

- *The Origin* Chapters 1, 2, and 3 and pages 712-727
- Wilson Chapter 1-9 and 20
- Winston*, The Biology of the Honey Bee* Chapters 1 and 4
- [Download Estoup et al. 1994.](https://dominicanu.instructure.com/courses/1703918/files/199608701/download?download_frd=1)

In addition, depending on the topic you choose, you may use any of the following:

- The Bill Moyers video from Week 1
- One or both of the two videos from this week, *The Silence of the Bees* and *The Evolution of Intelligence*

The Biology of the Honey Bee Chapter 5 (Nest Architecture), Chapter 6 (Age-Related Activities of Workers), Chapter 7 (Other Worker Activities), Chapter 8 (The Chemical World of Honey Bees), Chapter 9 (Communication and Orientation, or Chapter 10 (The Collection of Food)

• The USDA Report on the Stakeholders Conference on Honey Bee Health

• "Honey Bees as a Model for Vision, Perception, and Cognition."

• "Anti-social bees share genetic profile with people with autism" Links to an external site. and "Deep evolutionary conservation of autism-related genes"

"Bees Love Getting High on Caffeine" Links to an external site. and "Caffeine in floral nectar enhances a pollinator's memory of reward"

You may also use other sources that you identify through research; however, we do not expect you to do extensive additional research; the sources listed above will be sufficient for this assignment.

Here is a possible outline for your presentation:

- Slide 1 - Title, presenters, date
- Slide 2 - Brief description of the topic
- Slide 3 - Research question
- Slides 4-5 - Background about the issue
- Slides 6-9 - Literature Review
- Slide 10 - Passing the baton passage
- Slides 11-14 - Research proposal (the proposal, the material and equipment, the procedures)
- Slides 15-16 - Expectations about findings and alternative possibilities
- Slides 17-18 - Potential implications of the research (for bees, for the environment, for humans, for evolutionary science)
- Slide 19 - Invite questions
- Slide 20 - Thank You
- Slide 21 - References

We will provide sample Assignment 1 presentations.  Please let us know if you have any questions about them.

Grading Criteria

Research Question - 15 points.  The research question is clear, specific, interesting, capable of being answered through microsatellite analysis, and substantively connected to existing knowledge, prior research, and a knowledge gap of some kind.

Background, Literature Review, and Passing the Baton Passage - 20 points.   The background clearly and concisely identifies the context, history, and significance of the issue.  The literature review helps the audience understand the context for and significance of your research proposal.  The Passing the Baton passage makes it clear what gap in knowledge your research aims to fill.

Research Proposal - 25 points.  The proposal clearly identifies the goals of the research, the materials needed, the method that will be used, and the specific steps that will be carried out.

Expectations about Findings and Alternative Possibilities - 10 points.    Plausible expectations are clearly identified and substantively explained.  Plausible, significant alternative possibilities are identified and explained.

Potential Implications of the Research - 10 points.  Potential implications of the research for understanding honey bee behavior, for honey bee health, for humans, for the environment, or for evolutionary science are clearly identified.

Effectiveness of the slides - 10 points.  The slides are well designed, visually clear and appealing, and easy to read.  The text on the slides is written correctly, clearly, and concisely.  Visuals are appropriate and effective.

Effectiveness of the delivery - 10 points.  The spoken part of the presentation is delivered in a professional, authoritative, and engaging way; with good eye contact and minimal if any reading; and with an effectively modulated and well-paced tone, with few or no fillers ("um," "you know," etc.).  The group introduces itself, and there are smooth transitions between one group member and the next.  Questions are invited at the end of the presentation and are addressed professionally and knowledgeably.

Schedule

Week 4 - In class and online, begin discussing the assignment, possible topics, possible research questions, and sources for the literature review.

Weeks 5-6 - Work online with your team online to draft the slides; in class, continue working on the slides and begin rehearsing the delivery.   As needed, schedule a team conference with the professors.

Week 7 - Work online to complete the slides.  Meet in person to rehearse.  As needed, schedule a team conference with the professors.  In class, deliver the presentation, with resounding success!

# Assignment 2 - Group Poster Proposal for a Campus/Community Sustainability Project

Background

In Assignment 1, you learned how to work with a team to research, focus, develop, contextualize, and explain a proposed research project and present the proposal in the form of a slide presentation.  For this assignment you will work in teams to research, focus, develop, contextualize, and explain a proposed community sustainability education/action project and present that proposal in the form of a poster.

Assignment

Drawing on the Unit 2 assigned readings and your own research and investigations, work with your team to develop a proposal for a project to help members of a community interact with the natural world in a way that promotes sustainable practices and helps develop their own understanding of the possibilities and limits of the natural world.  The community may include the Dominican campus/community but should also include another community that may be connected to Dominican either geographically or in some other way.  Your team will choose the community, explain your choice, and carefully consider best ways to approach and communicate with that community.  You will embody this proposal in a poster that you will present in a poster session in class, with the rest of the class and invited guests, ideally including members of that community, as your audience. Whatever your specific proposal, aim to present it in a way that takes into account the resources, needs, and particular characteristics of the community you have chosen.

Topic Suggestions - These are just the ideas that we brainstormed.  We welcome other ideas or variations on these.

1. Research the history, present status, and physical characteristics of the piece of land at the southeast corner of Division and Thatcher and propose a use for this land that would promote ecological awareness at Dominican and in the surrounding community.
2. Work with River Forest Sustainability Commission and West Cook Wild Ones to propose a plan to research the locations of honeybee colonies in the OPRF community as well as the populations of native/wild bees.  Include a proposal for a meeting—symposium, potluck dinner?—between local beekeepers and local promoters of native wildlife.
3. Work with the American Indian Center in Chicago to propose a collaborative project with Dominican that would promote ecological awareness at Dominican and in the Chicago community, perhaps focusing on the ecological and indigenous history of the Chicago area.
4. Work with the School of Education, the Summer Gifted and Talented Program, and Dominican’s Sustainability Coordinator to propose a two-week summer program on ecological awareness for grade school children, in which students will explore and reflect on Dominican’s native landscaping, apiary, greenhouse, vegetable garden, composting, and general flora and fauna.   A possible variation on this option is to work with the Care for Creation staff at the Sinsinawa Mound, the Sinsinawa Dominican Motherhouse, to create an ecological education program at the Mound in Sinsinawa, Wisconsin.
5. Work with the Office of Civic Learning to create a list of Community-Based Learning projects focusing on ecological awareness in Chicago and the surrounding suburbs.
6. Work with Brookfield Zoo and/or River Forest's Trailside Museum of Natural History to propose a project that helps educate people about human evolutionary history and the place of humans in ecosystems.
7. Research how Dominican might draw on the ways of knowing of a community of your choice to help educate people about the COVID-19 pandemic, drawing on evolutionary reasoning and ecological awareness to help people understand public health recommendations, including masking and vaccinations.
8. Research how Dominican might help a community at risk of natural disasters (such as wildfires, rising ocean-levels, drought, etc.) prepare for and understand natural disasters.  What are some cultural strengths of the community that you could drawn upon?  How would you use evolutionary reasoning to collaboratively educate the community?
9. Research the proposed Civilian Climate Corps currently being debated as part of Biden's Infrastructure Plan and make a proposal *either* about how the future CCC might incorporate the concept of multiple epistemologies, as defined and explicated in Bang and Medin, *or*about how Dominican might prepare students to participate in the CCC.
10. Research the newly proposed Ecology and Environment Studies program at the Vatican's Lateran University, and make a proposal for how Dominican might use the resources of this program to create its own Ecological/Sustainability Studies program, perhaps building in some way on the sophomore seminar common text, Pope Francis's *Laudato Si'*

Guidelines for Completing the Assignment

1.  All proposals should be as fully informed as possible.  However, we recognize that you have limited time to research the proposal and that the phenomena you are investigating are complexly interactive.  Therefore, we do not expect you to have all the answers, although we would like to you be aware of questions that would need to be answered before proceeding.  Your proposal should be based on the information that you were able to gather in the limited time that we had, information about both the topic itself and about best practices related to that topic, AND it should be based on your knowledge about the resources, needs, and values of the Dominican or other entities that you are working with, as well as your team's knowledge of and vision about evolutionary thinking and ecological awareness.

2.  Your team should develop a poster design that suits your proposal and your esthetic preferences.  We will provide a template file for making the poster.

[Download here](https://dominicanu.instructure.com/courses/1703918/files/199743481/download?download_frd=1)) as a general guideline, but you might also want to look at some other models for inspiration.  For example, the posters from past seminars would be very good models but are not the only possibilities.  At the bottom of this page you will find four possible models for a poster about the environment, followed by three possible models for a poster that presents a proposal (as opposed to the more conventional academic poster that presents a hypothesis, methods, data, findings, and conclusion).  Please feel free to search online for other models.

3.  In class we will discuss how to present your poster, but here are a website and a video that you might find helpful.

<http://www.unl.edu/gradstudies/current/news/presenting-research-poster>

Science presentation skills:

<https://www.youtube.com/watch?v=q6y43AdOitA>

Schedule

Week 6 - In class, consider and ask questions about the assignment, including the suggested topics .

Week 7 - Outside of class, read the assigned academic articles and chapters from Wilson, Darwin, and *Anthill* and begin discussing with your team which topic you might choose and how you might address that topic.  In class, learn more about ecological awareness and discuss with your team plans, a schedule, and task assignments for completing the poster during Weeks 8 and 9.

Weeks 8 - 10 - Outside of class, continue researching your topic and working on your poster.  Post ideas, plans, and questions in Canvas discussions.  Schedule a conference with us if you have any questions at all.  Create a draft of your poster.  In class, get feedback on your poster draft, work on revisions, and begin practicing your poster presentation.

Week 11 - Outside of class, make final revisions to the poster and continue practicing the presentation; meet with us in conference if needed.

Friday, November 12 - Present the poster to invited guests.

Week 11 Weekend - Make any necessary changes to the poster.

Monday, November 15 - Submit final draft of poster template.

# Assignment 3 - Research Proposal on the Evolution of the Human Experience of Free Will

**Background**

You are now ready to think about how to conduct a research project on one of the great mysteries of human life:  the human experience of having free will.  Here are some of the most important ways in which you have prepared for this.

**First**, you have learned the basic principles of **natural selection**.  **Second**, you have read and had discussions about several **evolutionary puzzles**, that is, traits or behaviors that it would seem could not or would not have evolved through natural selection, for example the hive-building behavior of honey bees, sterile castes of insects, infanticide, and pregnancy sickness.  **In particular**, through your thinking about honey bee behavior and other evolutionary puzzles, you have developed some understanding about **how complex traits and behaviors might have evolved from simpler ones**.  **Third**, you have read many chapters in Wilson about what we can learn from **evolutionary thinking about human behavior**.  **As a specific example**, you have read about how an undergraduate student conducted multi-disciplinary **secondary research** on a profoundly puzzling phenomenon--human laughter--and synthesized this research by using an evolutionary framework. **Fourth**, you have learned how to read, understand, and analyze **academic articles**.   **In addition**, from a variety of course activities, you have begun to learn how to identify a **hypothesis**, analyze the **evidence** used to support it, consider how it might be either further **supported or challenged**, and develop your **own hypothesis** within that context.  Perhaps **most importantly**, you have learned how to **identify an unanswered question** in a line of research; do **secondary research** on this question; present this secondary research in the form of a **literature review**; identify a specific **gap in knowledge** on the basis of this literature review; describe a **research project** to address this gap, drawing on your knowledge about and experiences with a particular kind of **primary research**; and discuss the expected findings, confounding or limiting factors, possible alternative findings, and potential implications of the research proposed; **in other words, you have learned how to create a research proposal.**

In addition, by the time you are ready to draft your proposal, you will have learned the **basic definitions of and arguments about free will**, read and carefully analyzed an academic article investigating **how the subjective experience of having free will might have evolved** in humans, and had a chance to ask a psychologist and neuroscientist, a philosopher, and a theologian about **how different disciplines approach questions related to free will.**

Of course, since this is a final and culminating assignment, you will also be building on what you have learned so far and taking it to the next level.  For example, this assignment will ask you to consider how complex questions about evolution might be investigated from the perspective of different disciplines and to **think deeply about how the knowledge and methods of your own discipline**, or a discipline you have studied in one of your courses, might be used to probe a hypothesis about the human subjective experience of having free will.

**Assignment**

After carefully reading and discussing a variety of articles, both assigned by us and found through your own research, about the human experience of free will, write an **8-12 page proposal for a research project that probes**--gathers evidence that might support or challenge--the hypothesis offered by Michael Rose in **"Darwinian Evolution of Free Will and Spiritual Experience”** about the evolution of the subjective human experience of having free will.

The proposal should include an **introduction** that contextualizes the issue, including an accurate and succinct account of Rose's argument; a **literature review** that explains what has already been thought, argued, and discovered about the issue, including Rose's argument; a **proposal** that describes a specific research project designed to probe--that is, gather evidence to support or challenge--Rose's hypothesis; and a **discussion** of the anticipated findings, potential limiting or confounding factors, possible alternative findings, and potential implications of the research.

It should propose to **probe a specific aspect of Rose's hypothesis** using **any kind of disciplinary research**--e.g. biological, psychological, anthropological, ethological, historical, sociological, philosophical, or theological--that could produce **evidence that might credibly support or challenge** Rose's hypothesis.  Ideally, you should choose a kind of **research that you are already familiar with** from one or more of your courses, either through reading articles or carrying out the research yourself.

**Six Guidelines for Completing the Assignment**

1.  The proposal should **focus specifically on Rose's argument about how the subjective human experience of having free will might have evolved**, targeting a specific aspect of Rose's hypothesis, which we will be discussing in detail online and in class.

The proposal should **not** focus on whether or not free will exists, though this is a complex and fascinating question that you will be expected to have a basic grasp of.  You can get a quick overview of it:

https://www.livescience.com/19213-free-fate.html

read a more in-depth article about it:

https://www.theatlantic.com/magazine/archive/2016/06/theres-no-such-thing-as-free-will/480750/

or watch the short but informative video below, and we will also be discussing it in detail online and in class.

https://www.youtube.com/watch?v=O03oKmba0W4[Links to an external site.](https://youtu.be/O03oKmba0W4)

The following is a good podcast, *Rationally Speaking*, exploring free will:

http://rationallyspeakingpodcast.org/39-the-science-and-philosophy-of-free-will/

2.  In thinking about what kind of research you would like to propose, **consider talking to a professor in your major department, or of a course you are taking**, about how that discipline--for example, neuroscience, psychology, philosophy, or theology--studies the question of free will and how it might approach the claims that Rose makes about the evolution of the subjective experience of free will.  Of course, we will also be happy to talk with you about it, and we will encourage you to schedule conferences with us at different points in the process.  We will also have three visitors to the class--a psychologist/neuroscientist, a philosopher, and a theologian--and we encourage you to talk to them as well.

3.  See the **Guidelines below** on **identifying a specific aspect of Rose’s hypothesis** that you might want to probe, as well as sample **research questions.**

4.  The proposal might be **organized** something like this:

- **Introduction** – 1-2 pages.  Should include context about the issue, a succinct account of Rose's argument, and a one-sentence statement of the research question.
- **Literature Review** – 3-5 pages.  Should include relevant chapters from Darwin's The Origin; relevant chapters from Wilson, especially the later chapters, about group behavior, religion, and human evolution; possibly the short story that we will discuss in Week 10, “Please don’t tell me how the story ends”
- http://creationdemonstration.blogspot.com/2013/03/please-dont-tell-me-how-story-ends.html
- possibly the chapters that we read from Turtles All the Way;
- Definitely the Rose article itself and possibly any of the sources that Rose refers to; optionally any of the recommended but not required sources that we will post in the weekly discussions; your class notes from talks by our visitors from psychology, philosophy, and theology; and **at least three additional sources related to your specific research question**.  It should be organized appropriately to frame your research question, for example an examination of the topic that moves from broad discussions of it to specific, closely related research; a chronological account of how thinking and research on the topic has changed and developed over time; a discussion of the topic according to different aspects of it, perhaps showing how the proposed research will bring together these different aspects; or a discussion organized according to contrasting, even conflicting, perspectives.  A literature review might even combine organizational strategies.  For example, the **first part of the literature review** might focus on the idea of evolutionary puzzles, beginning broadly with Darwin's discussion of instincts as an evolutionary puzzle (Chapter 7, specifically the discussion of honey bee cell-construction), then moving on to Wilson's discussion of human evolutionary puzzles (e.g. pregnancy sickness), and then focusing specifically on the evolutionary puzzles of big brains, behavioral plasticity, and free will (Rose, guest panel, optional readings/podcast).  Then the **second part of the literature review**, in which you discuss the sources you have found in your secondary research, might be organized, depending on what you find and the story you want to tell about, as a debate, a history, or a puzzle with missing pieces.  **Remember that a literature review has a thesis of its own, which is essentially a brief overall description of the literature reviewed and which, ideally, points toward the gap in knowledge that the new or proposed research will address.** For example [the following are completely made up!] "The recent literature on evolutionary themes in spiritual experiences draws mainly on subjective descriptions of such experiences, but there are very few studies that use brain imaging to draw conclusions about what might cause spiritual experiences."  OR "Most research using evolutionary game theory focuses on environmental influences on the evolution of non-human animals."  OR  "The research on tool use in animals covers a wide range of species but does not investigate the evolutionary history of their tool use."  See #5, just below, for a more detailed discussion of literature reviews.
- **Taking the baton** – ½ page.  Should include a discussion of questions about or weaknesses in Rose's hypothesis and a brief description of how the proposed research that might help support or challenge the hypothesis.
- **Description of the research methods/procedures** – 1-2 pages.
- **Discussion Section** - 2-4 pages.
  - anticipated results - 1/2 to 1 page.
  - potential confounding/limiting factors - 1/2 to 1 page.
  - alternative possibilities – 1/2 to 1 page.
  - potential implications for understanding of human nature, contributions to evolutionary theory and to the specific discipline, and/or implications for social policy – 1/2 to 1 page.

5.  Your **literature review** should include about 10 sources, including chapters from Darwin and Wilson, the Rose article, notes from our panel discussion on free will, any of the optional sources that will be posted in Canvas discussions, and at least three sources specifically related to your own topic that you have identified through your research.

Remember that the most effective literature reviews are organized not simply by source but in some way that will help to frame your proposal, for example by different aspects of the topic, by different perspectives on a specific issue, or by the "funneling" method of beginning with a broad discussion of the issue and then focusing on more and more specifically-related research or discussions.  Remember also that 1. it is not necessary to give equal time to all sources, as you might do in an annotated bibliography; 2. it is perfectly fine to include ideas and information from one source in more than one part of the literature review, if for example one source contains ideas and information about different aspects of the topic; and 3. the literature review should help the reader see the relationships between the ideas and information in the different sources.  **Finally, remember that a literature review has a thesis of its own, which is essentially a brief overall description of the literature reviewed and which, ideally, points toward the gap in knowledge that the new or proposed research will address.** For example [the following are completely made up!] "The recent literature on evolutionary themes in spiritual experiences draws mainly on subjective descriptions of such experiences, but there are very few studies that use brain imaging to draw conclusions about what might cause spiritual experiences."  OR "Most research using evolutionary game theory focuses on environmental influences on the evolution of non-human animals."  OR  "The research on tool use in animals covers a wide range of species but does not investigate the evolutionary history of their tool use."

In writing the literature review, you should draw on your experience in putting together the literature review section of the presentation for Assignment 1, as well as any other experiences you have had reading or writing literature reviews. You might also want to look again at the literature review sections of Estoup. Bang & Medin, Root-Bernstein & Ladle, and the article by Matt Gervais Wilson discusses in Chapter 23, and also consult again any of the sources linked to below that you found useful for Assignment 1.

General Guidelines from the University of North Carolina Writing Center https://writingcenter.unc.edu/tips-and-tools/literature-reviews/

Guidelines for Literature Reviews Using APA style - includes links to other resources <https://research.lesley.edu/apa-6/literaturereview>

[Download Sample Literature Review in a published article: Robson, J., Francis, B., & Read, B. (2002). Writes of passage: stylistic features of male and female undergraduate history essays. *Journal of Further and Higher Education*, *26*(4), 351-362.](https://dominicanu.instructure.com/courses/1703918/files/199608891/download?download_frd=1)

Sample Literature Reviews Written by Undergraduate Students <https://library.famu.edu/c.php?g=577356&p=3982818>

**Please do not hesitate to consult with one or both of us at any point in the process of researching and writing the literature review!**

6. In writing the description of the proposed research and the specific discussions that follow it, you should also draw on your experience in the putting together the proposal and discussion sections of the presentation for Assignment 1, as well as any other experiences you have had reading or writing research proposals or the Methods and Discussion sections of research papers and article. You might also want to explore this collection of undergraduate student research proposals: <https://urop.uci.edu/journal/>

**Please do not hesitate to consult with one or both of us at any point in the process of writing the research proposal and discussion sections!**

**Schedule**

**Week 9** - Assignment introduced in class

**Weeks 10 and 11** - Read the assignment carefully; post ideas and questions in the Stage 3 discussions; and make an appointment to meet with us to discuss your focus, research question, and secondary research.

**Week 12** – Submit research question and brief annotated bibliography of the three or more additional sources that are specifically related to your research topic.

**Week 13** – Research questions and bibliographies returned with feedback; optional individual conferences on Monday and Tuesday.

**Week 14** – Draft of literature review and peer review of draft.

**Week 15** – Draft of completed proposal; Individual conferences to discuss the draft and our feedback.

**During final period** – Present proposals as part of a roundtable discussion about interdisciplinary inquiry into the subject of free will.

**Submit final draft in Canvas after presentation during finals period.**

## **Guidelines for Getting Started Thinking about a Research Question for Your Assignment 3 Proposal**

After looking over the assignment guidelines, do the following in preparation for getting started:  1. **identify a specific claim** made by Rose that you would like to probe with your research project (see examples below); 2.  **draft a specific research question** that could provide evidence that might either support or challenge the claim that you have chosen (see sample research questions on the other side of this sheet); and 3. **begin looking for prior research** (secondary sources) that would help you frame your research proposal.

**1.  Rose’s hypothesis about how the human experience of having free will might have evolved is built up from several specific, interlinked claims. Identify one of these claims that you would like to probe through your proposed research project.  Here are examples of such specific claims.  Feel free to use one of these or to choose another.**

A.  “The key step for the evolution of human free will occurred when our ancestral hominins developed tools that destabilized their evolutionary games. . . . This generated an arms race for spontaneous calculation of social behavior relatively unspecified by genetics . . . Naturally enough, a capacity for creative calculation that could be used against conspecifics [i.e., social benefits] could also be used against prey, or indeed or former predators [i.e., environmental benefits].  This unusual nexus of circumstances produced the mental arms race amplifier pattern of selection, which in turn led to our evolution of . . . Darwinian free will . . .” (78).

[Note that earlier in the chapter and then again on pages 78-79, Rose points out the apparent conundrum that, although humans have extensive behavioral flexibility that we experience subjectively as free will, in order to have survived as a species this behavioral flexibility must be held in check by the Darwinian imperatives of survival and reproduction, yet we don’t seem to make conscious everyday decisions on the basis of these imperatives.  The next claim that he makes is his attempt to resolve this conundrum.]

B.  “I propose [that] the brain operations that produce our subjective selves constitute only one of several major suites of integrating brain functions—our multiple executive camerae. More specifically, I propose that our experienced self is specifically the immediate tactical coordination center for our behavior. . . . [here he introduces the simile of the pilot vs. the captain of the ship].  We are not, in fact, free to choose the meaning of our lives, if the ‘we’ refers merely to the tactical center, or the pilot, which generates our subjective consciousness” (80).

C.  “. . . the hypothesis proposed here is that overall guidance is supplied by an elaborate, unconscious executive center that in fact directs us as to our ‘life destinations,’ even if it leaves the specific navigation to the piloting consciousness” (81).

D.  “Given this hypothesis of a supervisory Darwinian brain function, there should be detectable side effects of its operation, even if it is separate from conscious function. Spiritual experience provides abundant evidence for such side effects” (81). [Rose then identifies several aspects of this evidence; three examples are given below, E, F, and G.]

E.  “Although the diversity of spiritual experience precludes any simple characterization, such experience clearly points to aspects of human existence that are not usually straightforward or immediately accessible to consciousness, yet somehow concern the moral appraisal or ethical significance of our behavior” (81).

F.  “Additional evidence for the action of Darwinian supervisory functions in generating spiritual experience comes from cases when human brain function has been impaired by means of genetics, pharmacology, or extreme physiological stress. Under such conditions, many individuals experience contact with other ‘person(s)’ or ‘will(s)’ of some type” (82).

G.  “From the standpoint of multicameral Darwinian free will, a give-away feature of organized religions is that they characteristically offer strong claims or prescriptions concerning (a) prospects for immortality and (b) appropriate sexual conduct. And many religions and related ethical systems characteristically feature a component of what is broadly referred to as (c) ‘ancestor worship.’  All three of these things have strong Darwinian significance” (83).

H.  “‘Chimpanzees seem to display no acknowledgment of an identifiable and potent deity/being that directly affects their behavior’ [Tiger and McGuire, 2010, p. 206] . . .” (84).

**2.  Draft a specific research question that might provide evidence that would either support or challenge the claim that you have chosen. Here are examples of some research questions.  Feel free to use, adapt, or simply be inspired by one of these.**

- **To probe Claim A using principles and methods from biology:** Do other species that are known to use tools (some primates, certain birds) have categorizable greater behavioral flexibility than closely related species that have not been observed to use tools?
- **To probe Claim A using principles and methods from the social and behavioral sciences and business:** Does evolutionary game theory reliably predict Evolutionarily Stable Strategies involving complementary payoffs of social and environmental benefits of complex calculation ability?
- **To probe Claim B or C using principles and methods from neuroscience:** Does brain imaging reveal differences between subjects making decisions that could be classified as being related to survival and reproduction and (possibly the same) subjects making decisions not so clearly related to Darwinian ends?
- **To probe Claim D using principles and methods from neuroscience:** Does brain imaging reveal similarities between subjects making decisions that could be classified as being related to survival and reproduction and (possibly the same) subjects having experiences that could be classified as spiritual?
- **To probe Claim E using principles and methods from psychology:** Do mystics or experienced meditators report experiences that “concern the moral appraisal or ethical significance of our behavior”?
- **To probe Claim F using principles and methods from psychology:** Do subjects in which “brain function has been impaired by means of genetics, pharmacology, or extreme physiological stress” report experiencing contact with other “person(s)” or “will(s)”?
- **To probe Claim G using principles and methods from theology:** Does systematic sampling of religious texts (something like what Wilson describes in Chapters 28 and 29) support Rose's claim about the near-universality in religions of “strong claims or prescriptions concerning” prospects for immortality, appropriate sexual conduct, and ancestor worship?
- **To probe Claim H using principles and methods from biology or ethology:** Do any of the other great apes behave in ways that seem to suggest that their behavior is influenced by consciousness of spiritual presences?

**3.  Once you have tentatively decided on a research question, make an appointment with a librarian to decide on the best strategy for your secondary research, and in the meantime, do some preliminary searching in book catalogues and academic article databases, using appropriate key words.**

**Grading Criteria for Assignment 3**

**Introduction, including contextualizing discussion and statement of research question – 25 points**

**Accomplished -** Clearly and concisely provides information to allow the reader to understand the history and significance of the topic; provides a clear and helpful transition to the research question; and states the research question clearly.  **20-25 points**

**Mastering -** Provides information to allow the reader to understand the history and significance of the topic, but this may be confusing or unfocused in places; provides a transition to the research question; and states the research question.  **13-19 points**

**Beginning -** Does not provide enough information to allow the reader to understand the history and significance of the topic, or the information is confusing or unfocused; may not provide a transition to the research question; or may not state the research question clearly.   **7-12 points**

**Emerging -** Does not provide information to allow the reader to understand the history and significance of the topic; does not provide a transition to the research question; or does not state the research question clearly; or there is no introduction.   **0-6 points**

**Literature Review and Passing the Baton Passage – 100 points**

**Accomplished -** The literature review has about 10 sources, at least three of which are directly related to the research question; all of the sources provide useful context for the research question; they are described and organized in a way that helps the reader understand this context; and the passing the baton passage clearly summarizes the literature review, identifies a gap in knowledge or need for additional perspectives; and clearly and concisely explains how the proposed research project aims to meet this need.  **76-100 points**

**Mastering -** The literature review has about 10 sources, at least three of which are related to the research question; most of the sources provide useful context for the research question; there is an attempt describe and organize them in a way that helps the reader understand this context, but descriptions may be confusing or unfocused and the overall organization may lack a clear logic; the passing the baton passage summarizes the literature review, though the summary may be sketchy or unclear; attempts to identifies a gap in knowledge or need for additional perspectives, but this may be unclear; and explains how the proposed research project aims to meet this need. **51-75 points**

**Beginning -** The literature review may have fewer than 10 sources and/or may not have three that are related to the research question; and/or several of the sources do not provide useful context for the research question; and/or the description and organization of the sources do not clearly help the reader understand this context; and/or the passing the baton passage does not summarize the literature review and/or does not identify a gap in knowledge or need for additional perspectives and/or does not explain how the proposed research project aims to meet this need.  **26-50 points**

**Emerging -** The literature review has fewer than 10 sources and fewer than three that are related to the research question; many of the sources do not provide useful context for the research question; the description and organization of the sources do not clearly help the reader understand this context; and the passing the baton passage does not summarize the literature review, does not identify a gap in knowledge or need for additional perspectives, or does not explain how the proposed research project aims to meet this need; or there is no literature review and passing the baton passage.  **0-25 points**

**Proposal, including description of proposed research methods/procedures and discussions of anticipated results, confounding/limiting factors, possible alternative findings, and implications – 100 points**

**Accomplished -** Includes a detailed and clear description of proposed research methods/procedures, which are appropriate to the research question and distinctively imaginative; also includes well-informed, thorough, and thoughtful discussions of anticipated results, confounding/limiting factors, possible alternative findings, and implications.  **76-100 points**

**Mastering -** Includes a clear description of proposed research methods/procedures, which are appropriate to the research question; also includes well-informed discussions of anticipated results, confounding/limiting factors, possible alternative findings, and implications.   **51-75 points**

**Beginning -** The description of the proposed research methods/procedures is not clear and/or the methods/procedures are not appropriate to the research question; the discussions of anticipated results, confounding/limiting factors, possible alternative findings, and implications are unclear or undeveloped and/or parts of this discussion are missing.  **26-50 points**

**Emerging -** The description of the proposed research methods/procedures is not clear, the methods/procedures are not appropriate to the research question, or there is no adequate description of research method/procedures; the discussions of anticipated results, confounding/limiting factors, possible alternative findings, and implications are unclear or undeveloped, parts of this discussion are missing, or there is no discussion.  **0-25 points**

**Prose**

**Accomplished** **-** Sentences are correct, clear, and effective; word choice is distinctively precise; paragraphs are focused, developed, and coherent; transitions are cogent; and the in-text citations and list of references are complete, correct, and correctly formatted according to the appropriate citation system.  **20-25 points**

**Mastering -** Sentences are correct and clear; word choice is precise; paragraphs are focused and developed; transitions are appropriate; and the in-text citations and list of references are complete, correct, and correctly formatted according to the appropriate citation system.  **13-19 points**

**Emerging -** Sentences are mostly correct and clear, though there are some errors; word choice is sometimes imprecise or confusing; paragraphs may lack focus or development; transitions may be confusing or missing; and in-text citations and/or the list of references may be incomplete, incorrect, or incorrectly formatted.  **7-12 points**

**Beginning -** Many sentences are unclear or have errors; word choice is often imprecise or confusing; many paragraphs lack focus or development; transitions are often confusing or missing; and in-text citations and/or the list of references are incomplete, incorrect, incorrectly formatted, or missing.  **0-6 points**
